# A Munificent Bequest

**Published:** 1881-11

**Authors:** 


### A MUNIFICIENT BEQUEST.

Francesco Rizzoli, Professor of Surgery at the University of Bologna, who died recently, has bequeathed his vast wealth, estimated at nearly 6,000,000 francs, to the municipality of Bologna, with the stipulation that it is to be devoted to the completion and maintenance of the model Orthopedic Hospital on his estate at San Michele, in Bosco, an institution on which he had, during his lifetime, expended a sum of 2,000,000 francs.—*Lancet.*

